# Surveying and Monitoring for Vulnerability Assessment of an Ancient Building

**DOI:** 10.3390/s130809747

**Published:** 2013-07-31

**Authors:** Luigi Fregonese, Gaia Barbieri, Luigi Biolzi, Massimiliano Bocciarelli, Aronne Frigeri, Laura Taffurelli

**Affiliations:** Department of Architecture, Built Environment and Construction Engineering, ABC, Politecnico di Milano, via Ponzio, Milano 31-20133, Italy; E-Mails: barbierigaia@gmail.com (G.B.); luigi.biolzi@polimi.it (L.B.); massimiliano.bocciarelli@polimi.it (M.B.); aronne.f@gmail.com (A.F.); laura.taffurelli@polimi.it (L.T.)

**Keywords:** geometrical survey, monitoring, structural analysis, cultural heritage, terrestrial laser scanning, geometric leveling, topographic survey

## Abstract

This paper examines how surveying and monitoring improve our knowledge about ancient buildings, allow the interpretation of their structural response and help in the search for the best solutions for their conservation. The case study of *Palazzo del Capitano* in Mantua (Italy) is analyzed. In particular, the attention is focused on the use of a Terrestrial Laser Scanner (TLS) for surveying and monitoring too, considering that the building structural control has been performed in combination with other traditional topographic techniques such as geometric leveling and topographic networks for 3D control based on measurements through total stations. The study of TLS monitoring has been tested only in the last decade and it is an innovative method for the detection of displacements of particular surfaces. Till now the research has focused only on the use of TLS monitoring to control large structures and in particular landscape situations; thus its use for a civil construction and historical buildings is a new field of investigation. Despite the fact technological development and new methodologies seem offer new future potential for the analysis of ancient buildings, currently there are still important limits for the application of the investigated surveying and monitoring techniques.

## 1. Introduction

The increasing importance that current societies give to their historical and cultural heritage has promoted the drafting of specific legislations aimed at protecting and preserving it. In particular, Italian Codes [1,2] assert that the topographic techniques and their instruments provide an essential support to assess the structural response of an ancient building, for a conscious conservation. These Codes underline:

- the importance of having a precise geometrical survey in order to know the building in details. This is used in order to detect the anomalies and deformations of the buildings surfaces and, as a consequence, to set up an highly realistic structural 3D model for a Finite Elements Method (FEM) analysis;
- the importance of planning an appropriate monitoring program, based on the identification and interpretation of the buildings deficiencies. In this way it is possible both to control some significant parameters of the structures in time (crack movements, absolute or relative displacements of some points, rotation of walls or other elements) and to have a better understanding of their global structural behavior.

**Figure 1.**
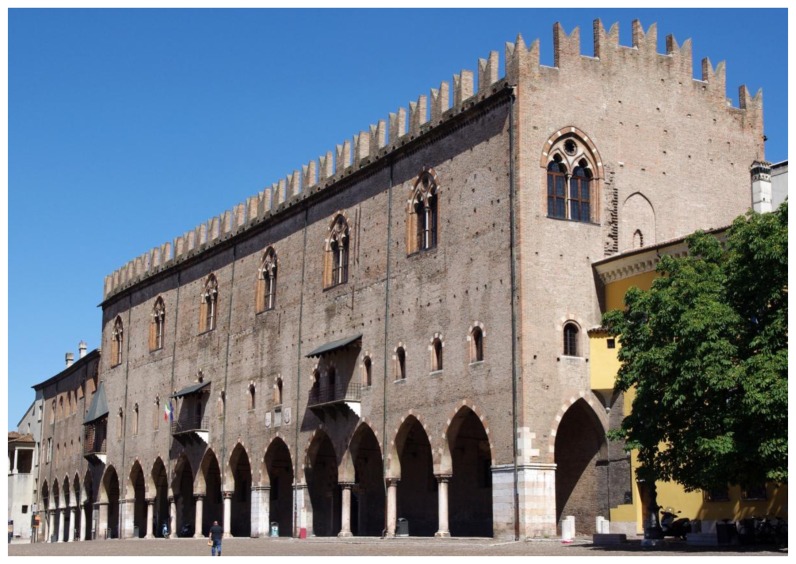
*Palazzo del Capitano* in Mantua. View from *Piazza Sordello*.

In the current paper, the contribution of the topographic techniques and instruments to the structural analysis of the cultural heritage was considered in relation to *Palazzo del Capitano*, one of the main masonry buildings of Mantua, Italy, see Figure 1.

This building was built between the last years of the thirteenth century and the first decade of the fourteenth century by the first Lords of Mantua. It is one of the most ancient parts of the *Palazzo Ducale* complex and over the centuries it has undergone a series of transformations which have partially changed its original structure. Since the eighteenth century, documents have shown the presence of significant out of plane displacements characterizing the two longitudinal façades overlooking *Piazza Sordello* and *Piazza Pallone*: they both present an inclination in the direction of *Piazza Pallone*. These static problems are probably due to ground settlement and they are related to the configuration of the structure and its transformations over time.

In the last two years a detailed structural analysis of the building—supported by a programmed monitoring campaign of the possible kinematic mechanisms that are still ongoing—has been conducted. This work was planned in order to analyze the structural response of the building and also to suggest a possible restoration.

The second section of the current paper deals with the geometrical survey in view of the structural analysis of *Palazzo del Capitano*. Before the structural analysis, the survey of the building was performed and completed through the integration of different techniques. The technology development in time allowed us firstly to use a direct and photogrammetric survey and secondly a laser scanner one. Thanks to this tool, the building was digitally acquired by means of dense point clouds which detected accurately the anomalies and deformations of the masonry walls. A 3D numerical model obtained was then used to perform the structural analysis. These allowed us to examine the building behavior both under serviceability conditions and in respect of exceptional events expected by current codes (such as an earthquake).

However, nowadays the potentialities opened by the TLS technique in relation to a new approach to the structural analysis have considerable limits. These are due to both the operative problems in large-size object acquisition and in data processing, and moreover to the transposition of the survey information in a numerical 3D model.

The third section of the paper deals with all the features related to the monitoring of *Palazzo del Capitano*. Simultaneously to the geometrical survey and structural analysis, the monitoring of the building was conducted for about one year with monthly or multimonthly cadences. Monitoring was aimed mainly at researching possible ground sinking or ongoing increases in the permanent out of plane displacements of the front façade overlooking *Piazza Sordello*. The periodic control of the movements in time allowed us to verify firstly the building structural behavior under normal operating conditions and secondly also under particular conditions that might have occurred during the period of observation.

In particular, in this paper, the attention has been focused to the use of the TLS for the control of the surfaces movements. Even in this case, the potential related to the use of such technique—which from a theoretic point of view allowed us a wide and very accurate control on a large number of points of the object under consideration—has clashed with the limits that are mainly related to problems of stability and precision of the georeferencing scans.

## 2. Geometrical Survey for Structural Modeling

The starting point for the knowledge of historical buildings belonging to the cultural heritage is the geometrical survey. Clearly, it must be supported by an in-depth historical and archival research and by thorough surveys on materials, structural techniques and geological aspects of the building site [1,2].

The integration of such surveys is essential to examine the buildings which, generally built before the twentieth century, are lacking in documentations about their construction, evolution and conservation. Most of these buildings were built when the common approach to design and construction was based on experience and empirical rules. Moreover, workers and available techniques in the past couldn’t control the realization of certain structures (especially of large sizes) with the precision that we can get nowadays. Often, time and historical events have damaged ancient buildings, with significant distresses, so that sometimes the buildings have changed or even distorted their originally structures.

In order to have a complete and thorough knowledge of the historical buildings it is essential to consider all the cognitive contributions provided by the geometrical survey. Indeed, it has to provide both the documentation and representation for the artistic and architectural aspects and the metric information for the structural analysis.

In particular, the ongoing development of the laser scanning technology allows us to reproduce accurately the three-dimensional geometry of all buildings acquired. In relation to the structural analysis, the TLS data can be used to reconstruct actual deformed configuration of the entire masonry surfaces of the buildings directly [3–5]. It can also used to provide the information needed for the elaboration of 3D models which allow us to investigate the expected behavior of the structure in particular conditions by means of FEM analyses [6–9].

### 2.1. Geometrical Survey of Palazzo del Capitano for the Structural Analysis: From Direct Survey to TLS

*Palazzo del Capitano* in Mantua rises three floors above ground, up to a height of about 22 m at the eaves line and 24.5 m at the ridge line of the gable roof. It has a rectangular plan of about 67–68.5 m × 16.5 m. Thicknesses of the bearing walls vary between 100 cm and 80 cm on the ground and at first floor, and between 80 cm and 70 cm on the second floor.

Of the two longitudinal façades, the north-west one overlooks *Piazza Sordello*—where there is the “public” side of the building—and the south-east one overlooks *Piazza Pallone*—where there is the “private” side, once overlooking the ancient *brolo*.

**Figure 2.**
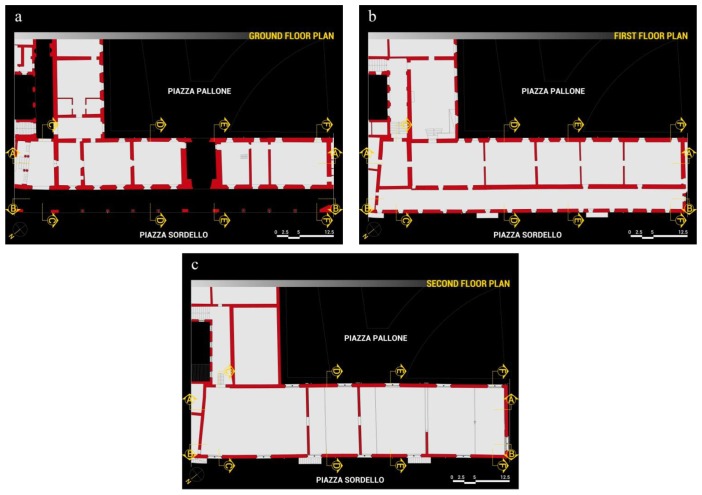
*Palazzo del Capitano* plans (**a**) Ground floor plan. (**b**) First floor plan. (**c**) Second floor plan.

**Figure 3.**
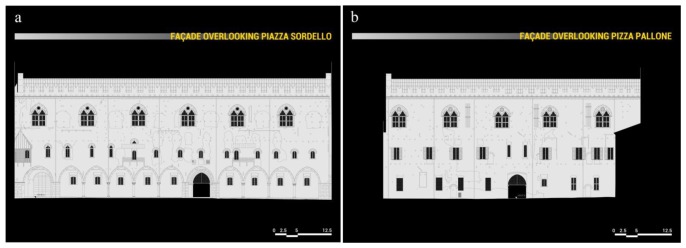
*Palazzo del Capitano* façades (**a**) façade overlooking *Piazza Sordello*. (**b**) façade overlooking *Piazza Pallone*.

**Figure 4.**
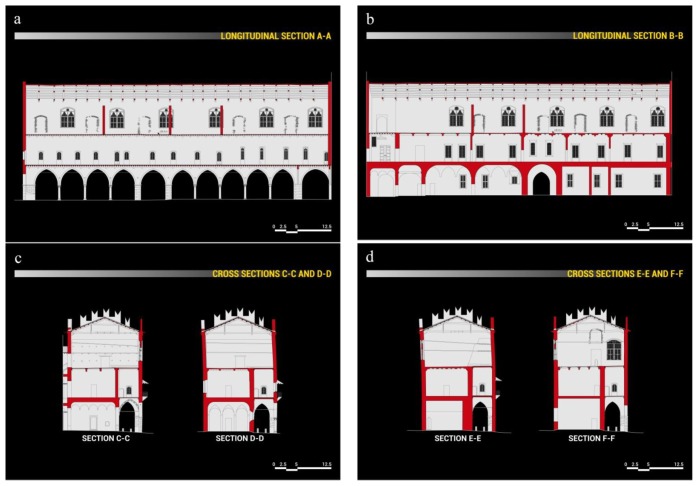
*Palazzo del Capitano* sections (**a**) Longitudinal section AA. (**b**) Longitudinal section BB. (**c**) Cross sections CC and DD. (**d**) Cross sections EE and FF.

The ground floor is characterized by a majestic portico on the *Piazza Sordello* side, and by a succession of rooms on the *Piazza Pallone* side. On the first floor, over the portico, there is a long hallway, known as *Galleria del Passerino*, while on the side overlooking *Piazza Pallone* there are the rooms of the Duke of Guastalla's apartment. The second floor is entirely taken up by the wide hall of the Armoury, known as *Salone dell'Armeria*, which is interposed by masonry partitions built at the beginning of the twentieth century to address the inclination of the longitudinal walls. The plans, façades and sections of the building in its current configuration are illustrated in Figure 2, Figure 3 and Figure 4.

The first surveys of *Palazzo del Capitano* were performed in the late sixteenth century, when architects of the palatine court drew up the plans of the building. However, since the early nineties of the twentieth century, the survey of the entire building in its current state has been performed using modern techniques and PC support, with different methods as required by the local superintendence for the historical and architectural heritage.

**Figure 5.**
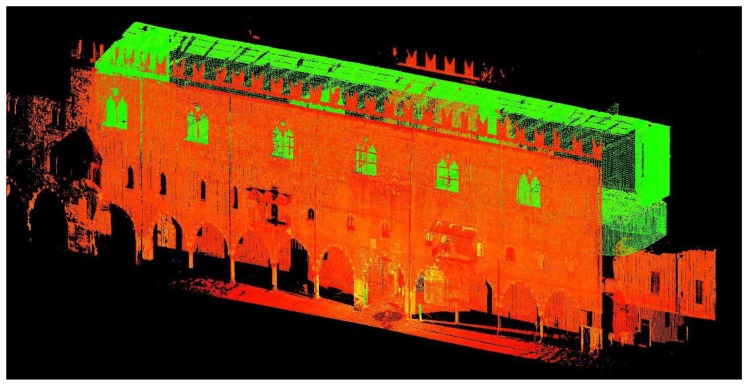
*Palazzo del Capitano* point cloud in Cyclone.

In particular:

- in 1993 a photogrammetric survey of the building façades and a direct survey of the internal rooms were performed. The plans of all floors, the two fronts and a cross section were executed at a nominal 1:100 scale;
- in 2005 a photogrammetric survey of the building façades and the internal walls of the *Salone dell'Armeria* was performed. Vettorial restitutions and digital orthophotos at a nominal 1:50 scale were executed. The survey was performed by means of a Rollei 6006 camera with a 16 MB Phase One digital back. The photogrammetric takes were carried out by a 40 mm lens at a distance of approximately 10 m. The images had a standard deviation σ in acquisition phase of about 2.5 mm, which is lower than the Graphical Error E_GR_ allowed for a 1:50 nominal scale (E_GR_ = 0.2 × 50 = 10 mm). The orthophotos were made in PCI Geomatics software and an Average Standardized Residual RSA in a range of 2–3 mm in correspondence of the control points was obtained: this was compatible with the accuracy provided by the support topographic networks;
- between 2005 and 2007 a Laser Scanner survey of the building façades, the interior space of the *Salone dell'Armeria* and the rooms of the Duke of Guastalla's apartment at the first floor was performed, see Figure 5. The survey was conducted using Leica HDS3000 and Leica HDS6000 laser scanners, especially to investigate the magnitude of the wall inclinations in a very detailed way;
- between 2011 and 2012 direct measurements were performed to complete the architectonic survey of the ground floor rooms and of the *Galleria del Passerino* at the first floor plan. The survey consisted of concatenated trilaterated quadrangles (six planimetric observations), framed by topographic control points. The relative tolerance error T of the 1:50 scale was assumed equal to 2.5 times the E_GR_ (T = ±25mm).

The integration of the surveying methods at different accuracy level was performed considering the TLS data as the primary source of the survey. The missing parts of the TLS data were ultimately obtained from the photogrammetric survey and direct measurements. However, thanks to the point cloud georeferencing, the future digital acquisition of the parts of the building not yet examined with the laser technique will allow us to further refine the geometrical survey.

TLS data were processed in Cyclone and then in CAD with the CloudWorks application. In Cyclone the scans were filtered and registered in a georeferenced system. In CAD they were supplemented with the direct and photogrammetric survey and then they were elaborated to evaluate the entity of the structural distresses along the entire surface of the building.

The outcomes of the geometrical survey show clearly the permanent deformation state of the longitudinal façades. The inclination begins in the upper part of the first floor and it increases more or less linearly as a function of the height. This state of deformation is probably due to ground settlement (and non uniform distribution of the soil properties) and to the lack of constraints joining the longitudinal façades to transversal walls. In fact, the wooden floors don’t establish a mechanical constraint in the plane and on the second floor of the *Salone dell'Armeria*, the tie-rods and bracing walls (which aren’t toothed to the perimetral walls) were inserted only after the detection of the permanent displacements.

**Figure 6.**
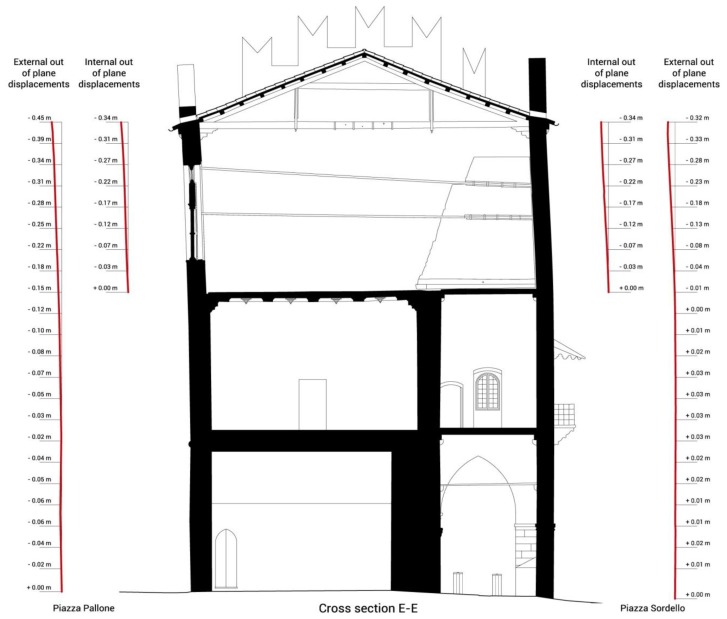
Cross section EE with the indication of the out of plane displacements of the walls as result by the Laser Scanner acquisition.

TLS data show that the main deformation occurs in the central part at the roof level. The façade overlooking *Piazza Sordello* presents an out of plane displacement ranging from 0.36 m to 0.38 m (from 4.4% to 4.6% of the last storey height), while the other façade overlooking *Piazza Pallone* presents an out of plane displacement from 0.32 m to 0.40 m (from 3.9% to 4.9% of the last storey height), see Figure 6. Finally, an inclination of the floor structure at the level of the *Salone dell'Armeria* of about 0.8% from *Piazza Sordello* (upper level) to *Piazza Pallone* (lower level) has been measured.

From some sections made at different heights of the building, a numerical model was realized in 3D-CAD in order to reproduce the actual geometrical configuration in detail, despite the simplifications adopted for some architectonical elements.

### 2.2. FEM Analysis of Palazzo del Capitano

**Figure 7.**
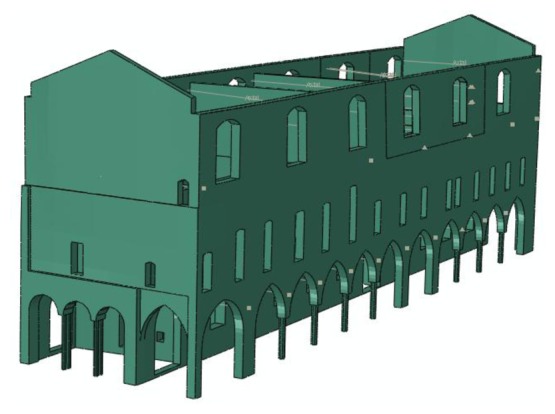
Geometrical model of the building in its current state. Lines represent the steel tie-rods.

The 3D-CAD geometrical model of the existing deformed configuration, including also the permanent out of plane displacements, was imported into the finite element program Abaqus [10], see Figure 7, and then discretized by four nodes tetrahedral elements for a total number of about 250,000 degrees of freedom. According to experimental measurements at various points of the masonry structure by means of double flat jacks, the average elastic modulus of the ancient masonry was 1,640 MPa. The compressive strength of the material was assumed equal to 2.4 MPa as suggested by the Italian Code [1] in Annex C8A.2.

**Figure 8.**
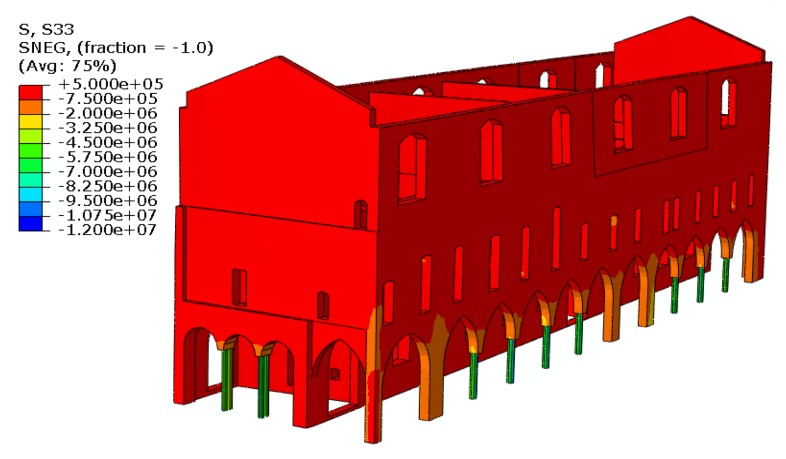
Vertical stress state (expressed in [N/m^2^]) in the longitudinal façade induced by the self weight of the structure.

This model was adopted to assess the seismic vulnerability of the structure [11]. In Figure 8 the vertical stress component due to the self weight of the structure are shown. The structure is compressed almost everywhere and only in a few points the analysis detects the presence of tensile stresses, which however are small enough not to lead to the partition of the section. The stress field due to the vertical loads is significant in some points: the highest compressive stress in the masonry walls is registered on the front of *Piazza Sordello*, near the springers of the arches, due to the strong reduction of the resistant section.

### 2.3. Limits and Potentialities of the TLS Geometrical Survey for the Structural Analysis of an Historical Building

From a theoretical point of view, the TLS geometrical survey has great potential, mainly thanks to the accuracy and speed in the data acquisition. The development of the TLS technique allows us to pass from a two-dimensional to a three-dimensional knowledge of the building under investigation. At the beginning, the building is detected only in correspondence of a few sections extracted by the direct measurements of a few remarkable points, later it is digitally acquired by a dense point cloud which describes the structure geometry continuously, including all the irregularities and anomalies of the surfaces.

These potentialities are important even when the geometrical survey is adopted for structural analysis purposes of the historical buildings. At the same level of knowledge of other factors, if the survey accuracy increases (and then it is possible a more accurate three-dimensional model), it is possible to obtain more reliable outcomes from the structural analysis.

However, the use of a laser scanner survey is related to a series of limits and problems that can’t be underestimated.

First of all, it is necessary to consider the difficulties normally related to the digital acquisition of an entire building (especially if it has important dimensions, such as *Palazzo del Capitano*) and to the data elaboration in order to obtain a numerical 3D model. In particular:

- the presence of obstacles interfering with digital acquisition of the building—such as vegetation, furniture or people walking while the laser scanner is operating;
- instrument capability, related mainly to their range;
- data processing time in particular related to the point cloud filtering operations. In addition, with large-size building several scans to acquire the object as a whole are required.

Secondly, the limits of the structural software referring to the laser scanner use for a numerical model construction have to be considered. Indeed, a detailed 3D model of every architectural feature, should produce significant calculation complexities and unacceptable data processing time. As a consequence, it is necessary that the point cloud be transferred into a simplified model neglecting the secondary elements which don’t participate—or take part only minimally—in the overall structural behavior of the building.

## 3. Structural Monitoring

Monitoring plays a very important role in the diagnostic process of an historical building. Through monitoring instruments, the evolution (as a function of time and of environmental condition changes) of parameters that could be dangerous for the structural efficiency is controlled. In order to estimate the presence of ongoing permanent kinematic mechanisms, due to intrinsic factors and not to cyclical seasonal phenomena, it is important to establish a suitable period of observation, from 12 to 24 months. Moreover, by means of a trend analysis, the long-term behavior of the building can be analyzed. In this way, monitoring allows us to integrate and increase the knowledge of the structural efficiency of an ancient building which comes from the FEM analysis.

### 3.1. The Vertical Displacement Monitoring of Palazzo del Capitano Pillars and Columns: The High-Precision Geometric Leveling

The vertical displacement monitoring of *Palazzo del Capitano* aimed to evaluate the presence of possible ongoing ground settling. It was performed through the high-precision geometric leveling of the building pillars and columns. This monitoring technique has already been applied to several historical buildings methodically, such as the Leaning Tower in Pisa [12] and the *Basilica di San Marco* in Venice [13].

#### 3.1.1. Planning and Execution of the Leveling Network

The high-precision geometric leveling of *Palazzo del Capitano* was realized in six observation campaigns between September 2011 and October 2012. The elevation changes of the object points in time were defined referring to the altitude of “point 0” (the reference point supposed fixed), placed in the center of *Piazza Sordello* where the bench mark of the Military Geographic Institute IGM95 is located.

**Figure 9.**
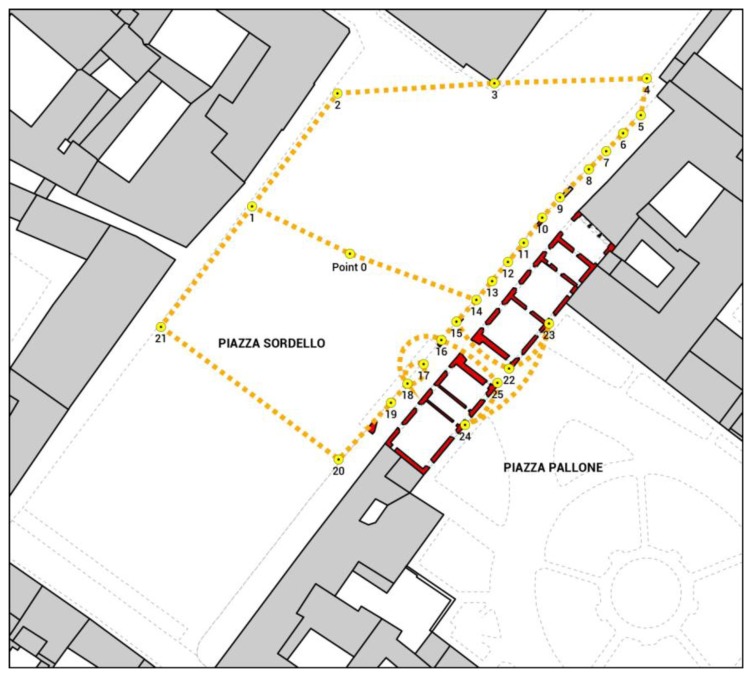
Plan of the leveling network with the localization of benchmarks and line leveling.

Monitoring was performed by a network made up with 26 object points and was structured into three closed loop lines leveling, see Figure 9.

As far as monumentation is concerned, the object points were materialized with stainless steel studs (for points situated at a height lower than 1 m) and cross arms (for points situated at a height above 1 m) sealed on the structures by a bi-component easy-removal paste. In the high-precision geometric leveling it is important that all points must be well tied with the structure, otherwise recorded displacements might represent monumentation displacements instead of structure displacements.

The points were located on the exterior structures of *Palazzo del Capitano* (exterior walls and an ancient door metal hinges of the façade overlooking *Piazza Pallone*, columns basements and pillars summits of the façade overlooking *Piazza Sordello*), on some remarkable elements in *Piazza Sordello* (mainly in correspondence with some lampposts) and on the columns of the historical *Magna Domus*.

The monitoring was performed by means of rigid invar staffs with bar code reading and a Leica DNA03 digital level, which has a standard deviation height measurement per 1 km double-run equal to 0.3 mm and a range for electronic measurements from 1.8 m to 110 m [14]. The detection technique used is known as “backward-forward, forward-backward”.

#### 3.1.2. Outcomes of the High-Precision Geometric Leveling

The results of the leveling data—processed through the Leica GeoOffice software combined with the academic software LEV in order to compensate the observations through rigorous least squares minimization—point out the presence of real structural displacements. The error for each measurement σ_Δ_ is considered equal to ±0.1 − 0.2 mm and then the tolerance error T is approximately 2.5 times the σ_Δ_ (T = ±0.25 − 0.50 mm).

**Figure 10.**
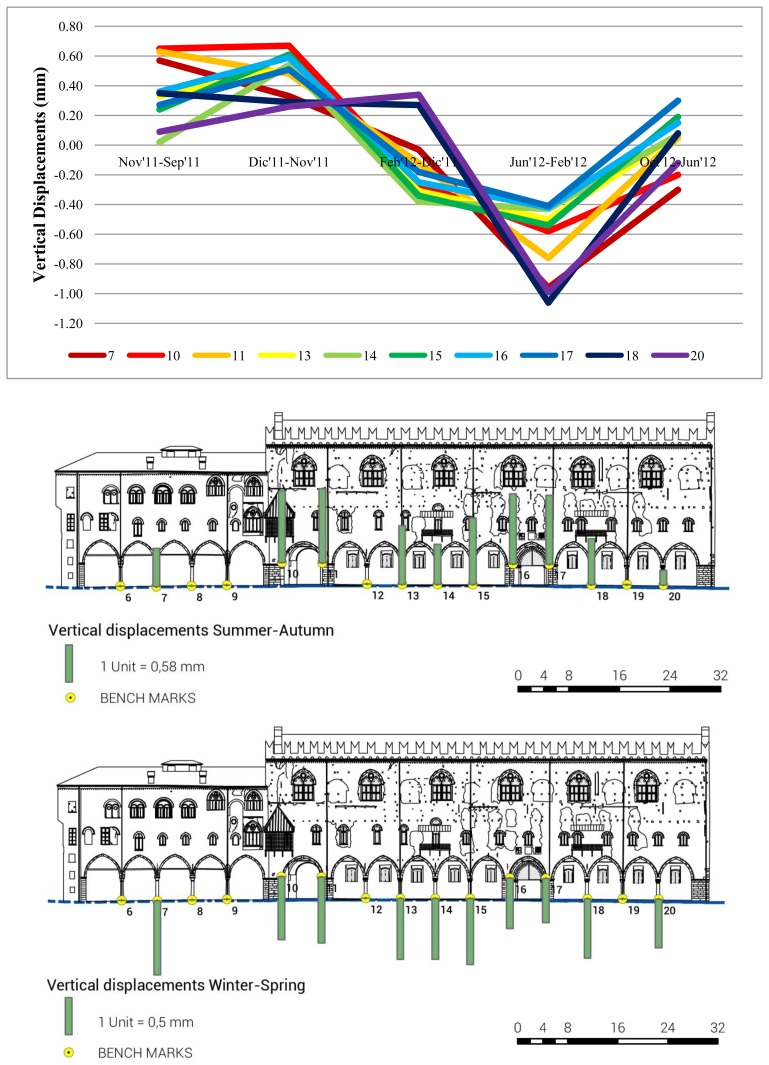

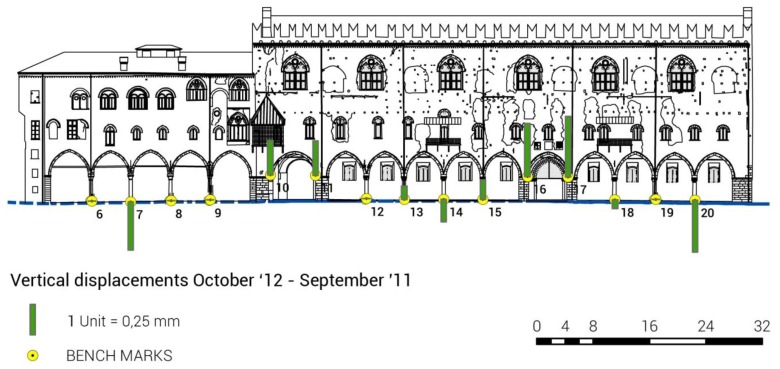
Evolution of the vertical displacements of the *Palazzo del Capitano* and *Magna Domus* benchmarks.

Table 1 and Figure 10 show that, on columns and pillars of the *Palazzo del Capitano* and *Magna Domus*, the data recorded a general increase of all points in summer (October 2012–June 2012) and autumn (December 2011–September 2011). Otherwise, in winter (February 2012–December 2011) and spring (June 2012–February 2012), the data recorded a general decrease of the points.

As a result, in one year, the vertical displacements tend to decrease generally to lower themselves. Only in the correspondence of the pillars (points 9–10 and 15–16) and of the outer side of the building (points 6 and 19), there are any significant elevation changes (values bigger than the tolerance T). For this reason, the displacement field appears due to a cyclical seasonal variations (thermal phenomena) and then there aren’t any sinking foundations, sub-pressures or elevation changes of the storage capacity.

#### 3.1.3. Limits and Potentialities of the High-Precision Geometric Leveling Technique

The high-precision geometric leveling is an excellent monitoring tool: it allows us to detect very small vertical displacements with a high degree of accuracy, thanks to the good control that the operator can exercise on the measuring operations. In the *Palazzo del Capitano*, the reliability of the results was also guaranteed by the site conditions: it was possible to carry out an effective distribution of the object points detected under the best conditions, as it was proved by the magnitude of the closing errors of the loop lines leveling network. The only real problem for the execution of the observation campaigns was due to some vandalism exercised by passers-by who, in time, have removed some studs, causing the loss of data on some columns.

### 3.2. The Horizontal Displacements Monitoring of Palazzo del Capitano Main Façade: From Total Station to TLS

The horizontal displacement monitoring of *Palazzo del Capitano* was aimed at evaluating the ongoing increases in the permanent out of plane displacements of the front façade overlooking *Piazza Sordello*. The horizontal displacements monitoring of the *Palazzo del Capitano* main façade was performed according to two different closely related approaches.

First of all, a Total Station topographic monitoring to detect displacements of the spatial coordinates of 24 points, placed between the windows of the first and the second floor, was performed. This technique, ordinarily used for monitoring operations of any kind of structures (galleries, historic buildings and monuments, bulkheads, retaining walls, landslides, decks…), has subsequently paved the way for the TLS monitoring testing.

Differently from the Total Station topographic monitoring, which allows us to control only a limited number of well-identified points of the investigated surface, the monitoring with TLS could allow us to extend the control over large portions of the surface and, potentially, on the whole surface, which is clearly described by a dense point cloud acquired with the highest achievable accuracy.

The TLS technique for monitoring displacements adopts an area-based method. The deformation analysis results from a comparison between two scans acquired at different times by considering mathematical surfaces fitting measured points. In this way noise could be reduced. Given that the different point clouds have been already georeferenced to the same reference system, the comparison between interpolated surfaces allows us to evaluate displacements [15].

#### 3.2.1. Introduction of TLS Monitoring

The Laser Scanning technology for the measurement of structural deformation was adopted for the first time at the beginning of the twenty-first century. Previously, in fact, the supposed lack of accuracy of the TLS system in measurement of individual points had precluded its use for monitoring applications.

Gordon et al. [16] experimented with TLS to detect the surface deformations of wooden and concrete beams subjected to loads. They confirmed the reliability of the laser scanner results compared with the measurements acquired by means of photogrammetric techniques; in this way they proved that the theoretical accuracy of the TLS on a surface generated by a dense point cloud is much greater than the precision attended from the detection of a single point. The fitting surfaces generated by the approximation of the acquired points allow us to compensate the errors on individual points, improving the general accuracy of the data.

However, till now the TLS technique for monitoring displacements has been performed only for the control of large structures and for geological applications related to the landscape. It has been used in situations where problems of particular structures of the landscape might represent a serious danger for human life and might cause an immense damage in terms of economic losses. This is the case of the large water dams [15,17], locks [18,19], or landslides [20] and quarries [21]. Therefore, the TLS monitoring of an architectural structure belonging to the historical and artistic heritage is a new field of investigation.

Actually, in the last few years, some researchers have already tested the possibility of using the potential of laser scanning for deformation monitoring of historical buildings. In 2009, Van Genechten *et al.* [22] published the results of the tests to evaluate the accuracy of TLS monitoring in detecting the deformations induced on a masonry arch. However, in this case, the experiments were performed in laboratory, under ideal conditions. Currently, the existing literature reports that the use of laser scanning for deformation monitoring of structures has actual utility for the detection of seasonal deformations of points recording displacements in the range of few centimeters [23]; this is what is expected in the case of *Palazzo del Capitano*. In other cases, a possible way of monitoring very complex architecture—integrating different instrumentation and modeling methods—is permitted by the use of TLS instrumentation [24]. The development that this process will support will be the transformation from model to 3D BIM product for structural analysis [25].

#### 3.2.2. Planning and Execution of the Topographic Network and Laser Scanning Monitoring

**Figure 11.**
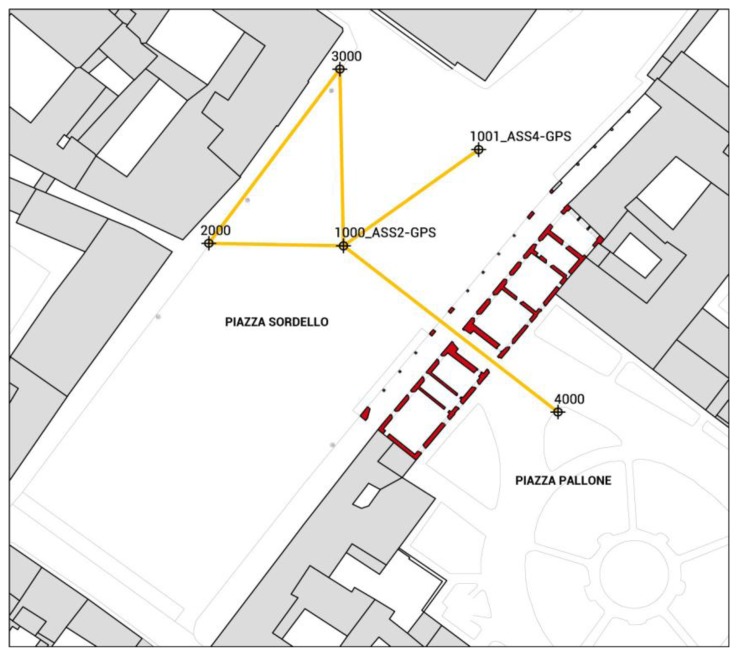
Plan of the topographic network with the localization of vertices.

The monitoring of the main façade of the building was realized in seven observation campaigns between October 2011 and October 2012. The topographic monitoring was performed with a Leica TCRA1203 Total Station (a particular model of the Leica TPS1200+). Data georeferencing in the ETRF2000 Reference System (RS) with UTM projection was performed by a rigid roto-translation with respect to the vertex 1000 and the direction defined by the vertices 1000–1001 (in this way the modulus of linear deformation is always equal to 1). These two points, placed in *Piazza Sordello*, have known coordinates identified by the IGM national network (point 1000 is in correspondence of the ASS2-GPS and point 1001 is in correspondence of the point ASS4-GPS). Then, from this reference system, the direction 1000–2000 was defined and it has been considered fixed in the following stage of topographic analysis for the definition of measurements with the Total Station starting from the vertices 1000, 2000, 3000 and 4000, leaving out the 1001 vertex, see Figure 11. The station vertices were implemented with steel studs sealed on the paving.

The points of detail, measured from the vertices, were arranged on the building façade, for the displacement monitoring, and around the squares, for georeferencing system. The collimation of the network vertices has been realized by means of mini reflective prisms which have been collimated with infrared remote measure setting (IR-Mode) and the automatic target recognition.

**Figure 12.**
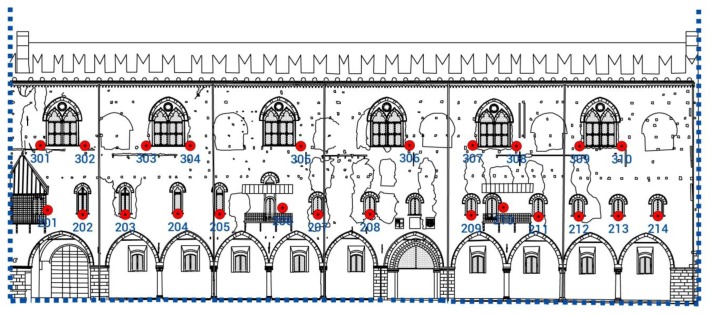
Individuation of the points of detail detected with Total Station on the *Palazzo del Capitano* façade overlooking *Piazza Sordello*.

The 24 control (CTRL) points on the building façade, were identified by plasticized reflecting square shapes (4 × 4 cm) sealed by a bi-component easy-removal paste on the sides of the windows that can be opened, see Figure 12. CTRL points were collimated with IR-Mode, without the automatic target recognition because of the distance of the points from the Total Station. In standard mode, the IR-Mode accuracy (standard deviation σ) is equal to 1 mm and the distance measurement range for mini prism is 1,200 m and for reflective tape is 250 m [14].

The points of detail for cloud georeferencing (TLS points) were identified by paper targets that were placed and then removed during several observation campaigns. TLS points were collimated with reflectorless remote measure setting (RL-Mode), with a PinPointer R400. For distances shorter than 500 m, the RL-Mode accuracy (standard deviation σ) is equal to 2 mm [14]. Processing data points software (Cyclone) recognizes TLS target vertices automatically.

Every point was detected by the Total Station twice (with both direct and reversed modes) in order to eliminate instrumental errors and increase precision.

At every observation campaign, the TLS monitoring was performed placing the laser scanner in the center of *Piazza Sordello*, in correspondence of the IGM point ASS2-GPS: in this way it was in a central position with respect to the building façade, at a distance of about 28 m from the median line of the front plane.

The compact Leica HDS6000 Laser Scanner in the first campaign and the next model Leica HDS7000in the following campaigns were used, respectively.. Both models are phase-based, dual axis compensated, ultra-high speed laser scanners, with survey-grade accuracy and field-of-view. In particular, the HDS6000 has a range up to 79 m, a scan rate up to 500,000 points/s, a modeled surface precision/noise at 25 m between 2.0–3.0 mm and at 50 m between 4.0–7.0 mm and 40,000 points/360° detected in “ultra high” scan resolution. The HDS7000 has a range up to 187 m, a scan rate up to 1,016,727 points/s, a range noise at 25 m between 0.5–1.0 mm and at 50 m between 0.8–2.7 mm and until 100,000 points/360° detected in “extremly high” scan resolution [14].

To eliminate the noises that could have been caused by the superimposition of multiple scans, a single scan, that included the entire façade of the *Palazzo del Capitano* overlooking *Piazza Sordello*, was performed. In this way the acquired TLS data were more defined. Scans were set at the highest resolution possible, available time permitting. Both HDS6000 and HDS7000 Laser Scanners were set up with ultra-high scans resolution. In particular, the HDS7000 laser acquisitions were entirely performed with a normal quality, with windows in high quality only in correspondence to the TLS points, in order to obtain an higher precision in the recognition of targets in Cyclone and, as a consequence, in the point cloud georeferencing.

In particular, the registration errors of the TLS targets were lightly superior than the standard deviation σ of the points calculated by the least squares compensation of the topographic data; however they are in the range of tolerance error T (1σ = ±2 mm; T_(95%)_ = 2σ = ±4 mm; T_(99%)_ = 3σ = ±6 mm). As an example, the Table 2 show the registration errors obtained in the acquisition of February 2012.

**Figure 13.**
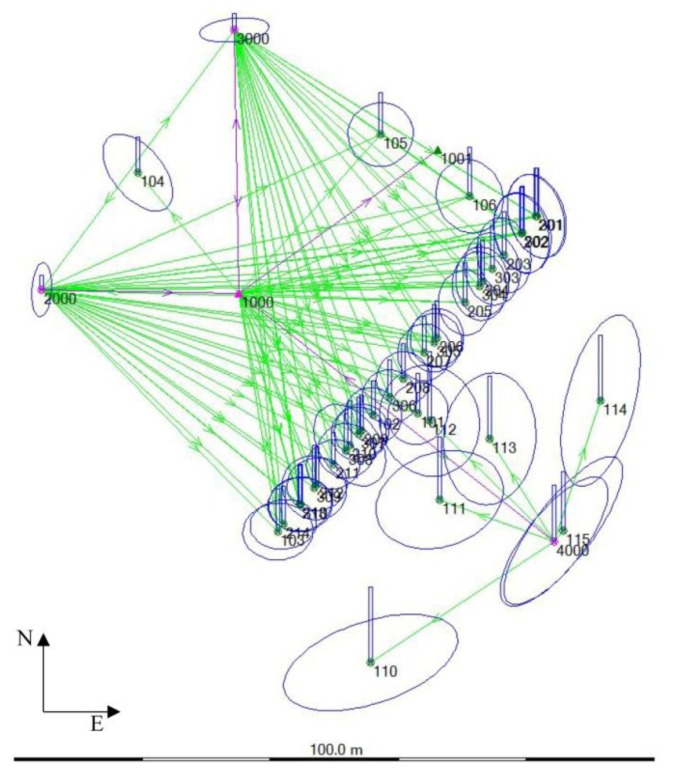
The October 2011 topographic network georeferenced in UTM-ETRF2000 RS. The magnification factor of the error ellipses compared with the network is equal to 5,000.

To perform the data analysis, it was necessary to change the RS to understand the horizontal displacements of the façade: in the UTM-ETRF2000 RS the façade plane was sloping with respect to the axes East-North (X-Y), see Figure 13.

**Figure 14.**
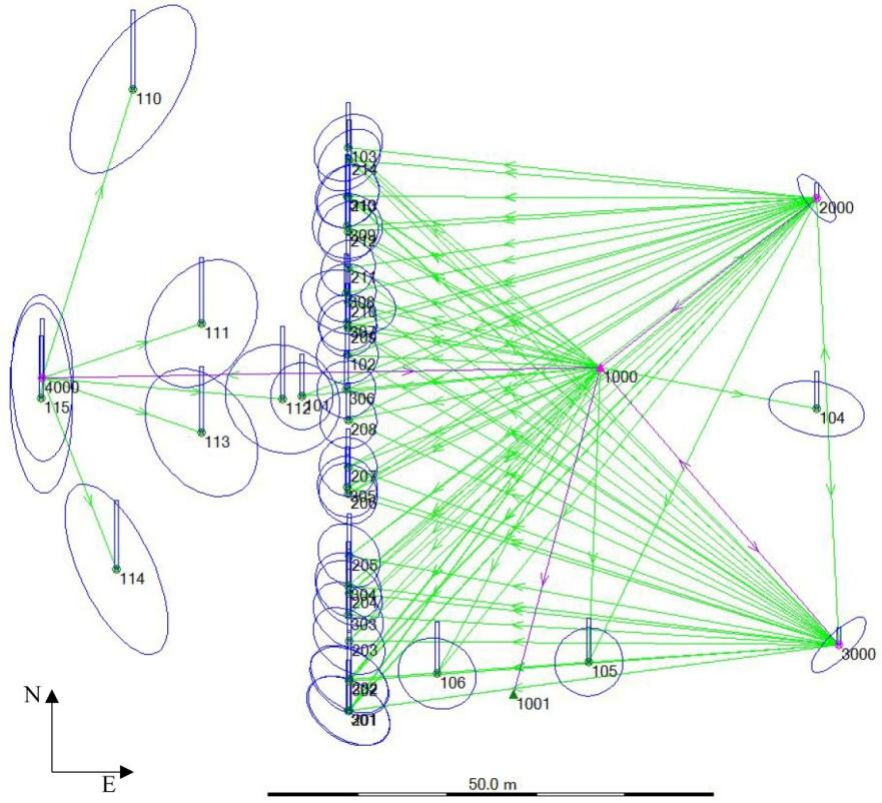
The October 2011 topographic network after the roto-translation of all points in a new 3D coordinate system. The magnification factor of the error ellipses compared with the network is equal to 5,000.

**Figure 15.**
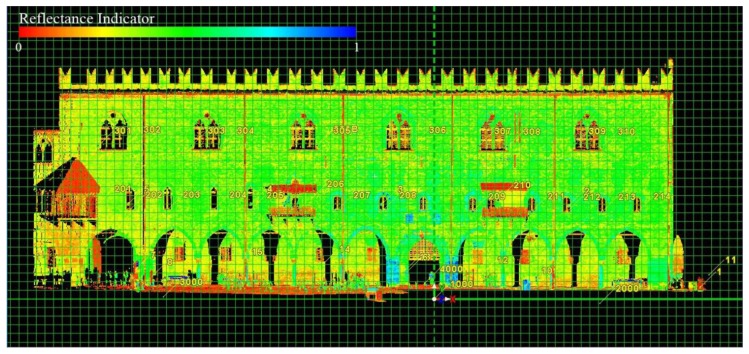
Cyclone. Point cloud of the *Palazzo del Capitano* façade in the new local reference system with the axes rotated. Hue expresses the reflectance of the surface materials acquired by the TLS.

In the new defined local 3D coordinate system, all points were rigidly roto-translated to have the building façade in a parallel position to the North direction (Y axis) and the point 1000 in the position (0,0,0). So the horizontal displacements were easily readable along the East direction (X axis), see Figure 14 and Table 3 and Table 4. Then, for processing data by means of 3D Analyst Tools in ArcGIS software, a rotation of the Cartesian axes was performed to have the building façade in the XY plane and the out of plane displacements readable along the Z axis (with direction from *Piazza Pallone* to *Piazza Sordello*), see Figure 15.

#### 3.2.3. Data Analysis of the Total Station Monitoring

From the Total Station monitoring of the CTRL points on the *Palazzo del Capitano* façade, the component of the displacement vector in the out of plane direction (Z axis) is analized.

In particular,:

- partial displacements are in the range of a few millimeters;
- total displacements generally tend to be withdrawn in the limits of the instrumental precision (σ = ±1 mm on average) evaluated with a tolerance T = ±2 mm. This would mean a general recovery of displacements detected in the partial measurements.

The outcomes of the topographic monitoring with Total Station in z direction are recorded in Table 5.

#### 3.2.4. Data Analysis of the Laser Scanning Monitoring

According to the area-based method, mathematical surfaces fitting measured points on the basis of the Inverse Distance Weighted (IDW) algorithm (raster grid with a constant mesh size) —through subtraction algebraic functions—were generated. Then, the displacements occurred in time were calculated in ArcGIS from two georeferenced scans acquired at two different times.

For data management reasons, the analysis was only performed on two portions of the masonry: two slices at two different heights of the façade with a thickness of 1 m and characterized by homogeneous masonry without discontinuities. The first one was placed above the windows of the second floor, the second one was placed between the first and second floors.

From the data analysis of the two slices under consideration, it is possible to observe:

- partial displacements are generally bigger than one centimeter (in the slices placed between the first and second floors, the partial displacements are in the range of +1.4/−2.1 cm);
- total displacements are calculated in the range of +7/−9 mm: this would mean the presence of a residual deformation of the masonry at the end of the entire observation period.

**Figure 16.**
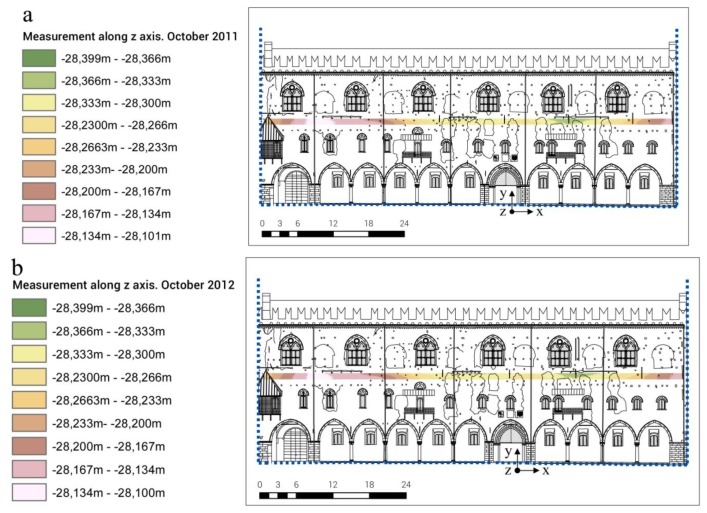
TLS data analysis in ArcGIS software: method of points interpolation based on the Inverse Distance Weighted (IDW) algorithm (**a**) October 2011. (**b**) October 2012.

**Figure 17.**
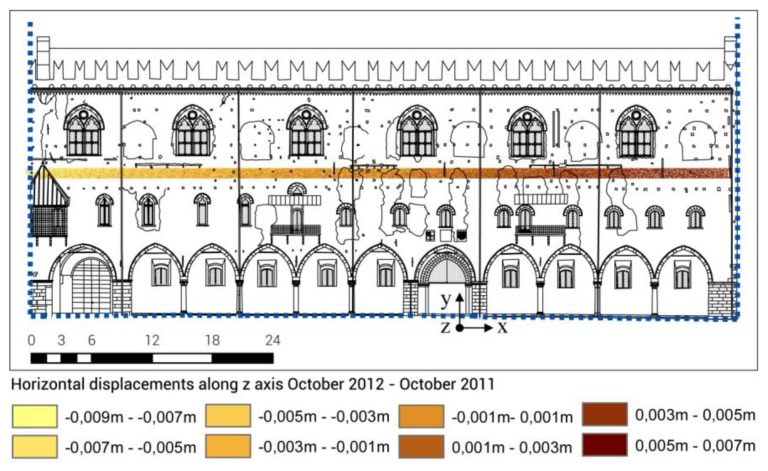
TLS data analysis in ArcGIS software: horizontal displacements calculation by means of the area-based method.

Figure 16 and Figure 17 show the data elaboration of the lower masonry slice analyzed, in relation to the total displacements.

#### 3.2.5. Synchronic Reading of the Results

From the comparative analysis of the monitoring results with Total Station and TLS, a still not exhaustive picture of the situation is evident. However, some reflections on results and procedures adopted can induce us to look for some data interpretations that may contribute to the future development and improvement of the tested monitoring techniques.

Generally, it is possible to observe that the deformation data detected with the two monitoring methods tend to diverge significantly. The Total Station monitoring records horizontal displacements in the range of a few millimeters; otherwise, the laser scanning monitoring records horizontal displacements in the range of few centimeters.

The main factor that could have affected the results is probably due to the possible inaccuracies in CTRL points acquisition with the Total Station. In particular:

- possible laying errors committed by the operator. As mentioned above, because of the distance between the network vertices and the targets on the building façade, the measurements were performed without automatic target recognition: this didn’t allow the operator to eliminate possible laying errors. In view of the magnitude of the displacements, even a small imprecision in centering the targets could have caused a significant error in data detection. However, now, there is no possibility to suppress this uncertainty;
- stability of the reference system. The existing literature on the use of laser scanning technology for structural monitoring states that the reliability of the deformations estimate is closely related to the accuracy of georeferencing operations. Certainly, in the case study under consideration, the materialization of a stable reference system was the first problem to influence the results accuracy. As above mentioned, in the *Palazzo del Capitano* case, the vertices of the topographic network were implemented with steel studs sealed on the paving, which were centered through the laser plumb of the total station. Under ideal conditions, it would be necessary to materialize the vertices through the provision of reinforced concrete plinths, on which rigidly fixing metal references to the precision keying of the metal pillars where running the forced centering with total station. These arrangements would guarantee the stability of the reference system. For example, the method described above, was performed for the deformations monitoring of the Cancano dam [15], of the landslides of two rock faces in the lombard Prealps [20], and for the control of the inclination of the Leaning Tower of Pisa. Otherwise, in the case study of the *Palazzo del Capitano*, the monumentation of the reference system wasn’t possible due to the constraints imposed by the local Superintendence for the Architectural Heritage, for the monumental complex of *Piazza Sordello*.

## 4. Conclusions

In this paper, some significant considerations about the combined use of surveying and monitoring as tools to increase the knowledge about an historical building were presented.

Firstly, it has been underlined the importance of having a geometrical survey that can represent in the most complete, accurate and truthful way an analyzed situation. The information provided by the geometrical survey are the basis for the definition of a reliable structural 3D model, adopted to simulate the expected behavior of the building in normal operating conditions and in respect of exceptional events according to the current codes.

In this contest, the potentialities offered by the use of a TLS to the survey of an historical building were evaluated. It was observed how this technology allows us to minutely and completely restore all the irregularities and anomalies of the acquired objects, enabling the construction of very realistic 3D models. Otherwise, problems related to the use of TLS data for the structural analysis were analyzed, too. These problems are mainly due to the way the metrical information can be transferred in a simplified 3D model, which is able to simulate realistically and in a useful and acceptable time the building structural behavior.

Secondly, the attention has been focused on the importance of monitoring to understand the real structural behavior of the building (and then recording the real displacements) in a medium-long term period. In this context different monitoring techniques have been integrated. Some of them have already been tested in time—high-precision geometric leveling to measure vertical displacements and topographic monitoring to measure horizontal displacements of some well-identified points in the main façade—others have yet to be tested—TLS monitoring to measure horizontal displacements of entire portions of the main façade. As it was often underlined, the TLS monitoring technique has already been applied for the control of movements in large structures and in particular landscape situations; however its application in civil structures has to be verified yet.

The experience with the *Palazzo del Capitano* is a starting point to test the efficiency of the laser scanner technique as a monitoring tool for historical buildings. Given that, on the territory investigated, there is a considerable number of building belonging to the national and international cultural heritage and given that significant seismic events occurred last year in the Mantua area, in the near future there will be the possibility to test thoroughly and systematically the real efficiency of the laser scanner technique for the control and the protection of civil structures of significant historical and cultural relevance. From the outcomes and the experience reported in the current paper, it is possible to attest to the methodology and rigor needed to encourage improvements in the TLS monitoring technique, in order to achieve more reliable results in the detection of the structural movements of the investigated buildings, in the future.

## Figures and Tables

**Table 1.** Partial and total vertical displacements of the leveling bench marks on the columns and pillars of *Palazzo del Capitano* and adjacent *Magna Domus*. Missing data are due to benchmark removal.

**Bench- Mark**	**Partial Displacements 11/11–09/11 (mm)**	**Partial Displacements 12/11–11/11 (mm)**	**Partial Displacements 02/12–12/11 (mm)**	**Partial Displacements 06/12–02/12 (mm)**	**Partial Displacements 10/12–06/12 (mm)**	**Total Displacements 10/12–09/11 (mm)**
5	0.65	0.35	−0.03	---	---	---
6	0.57	0.33	−0.03	−0.96	−0.30	−0.39
7	0.58	---	---	---	---	---
8	0.48	0.52	---	---	---	---
9	0.65	0.67	−0.26	−0.58	−0.20	0.28
10	0.63	0.48	−0.12	−0.76	0.05	0.28
11	0.54	---	---	---	---	---
12	0.33	0.52	−0.31	−0.50	0.07	0.11
13	0.02	0.54	−0.38	−0.43	0.07	−0.18
14	0.24	0.61	−0.34	−0.54	0.19	0.16
15	0.36	0.59	−0.25	−0.42	0.15	0.43
16	0.27	0.51	−0.18	−0.41	0.30	0.49
17	0.35	0.29	0.27	−1.06	0.08	−0.07
18	0.24	0.26	---	---	---	---
19	0.09	0.26	0.34	−0.99	−0.12	−0.42

**Table 2.** Registration errors recorded in the acquisition of February 2012.

**ID**	**Error Vector (m)**			**Error (m)**

	**x**	**y**	**z**	
1	0.002	−0.002	0.003	0.004
2	0.000	0.001	−0.006	0.006
3	0.001	−0.003	0.005	0.006
4	−0.004	0.001	0.001	0.004
5	0.001	−0.002	−0.001	0.003
6	0.002	0.003	−0.005	0.006
7	−0.002	0.001	0.003	0.004

**Table 3.** Coordinates and standard deviations of the points of detail detected on the *Palazzo del Capitano* façade. Data refer to October 2011 in new 3D coordinate system.

**Bench Mark**	**Coordinates (m)**			**Standard Deviations (m)**		
	
	**E**	**N**	**Elevation**	**E**	**N**	**Elevation**
201	971.8506	968.6920	33.3835	0.000592	0.000584	0.000528
202	971.8702	961.1654	32.6732	0.000579	0.000573	0.000508
203	971.8640	969.1189	32.6706	0.000566	0.000558	0.000487
204	971.8786	974.4860	32.7069	0.000554	0.000536	0.000463
205	971.8709	978.7395	32.6572	0.000552	0.000515	0.000446
206	971.7867	985.7351	33.9247	0.000557	0.000473	0.000430
207	971.8088	988.7880	32.6496	0.000566	0.000454	0.000414
208	971.7806	994.0845	32.6549	0.000578	0.000425	0.000405
209	971.7555	1,004.4292	32.5060	0.000583	0.000415	0.000403
210	971.7532	1,007.3552	33.8211	0.000966	0.000540	0.000563
211	971.7390	1,011.2715	32.4856	0.000573	0.000449	0.000415
212	971.7583	1,015.3628	32.4675	0.000566	0.000475	0.000427
213	971.8111	1,019.4217	32.4717	0.000563	0.000500	0.000441
214	971.8500	1,023.5099	32.4798	0.000564	0.000523	0.000458
301	971.7989	961.1570	40.1443	0.000590	0.000583	0.000548
302	971.8681	964.5062	40.2765	0.000577	0.000571	0.000533
303	971.8305	971.9479	40.1427	0.000555	0.000543	0.000504
304	971.8007	975.2991	40.1139	0.000548	0.000528	0.000493
305	971.6300	986.4897	40.1335	0.000547	0.000467	0.000469
306	971.6133	997.5203	40.1605	0.000562	0.000414	0.000461
307	971.6313	1,005.1214	40.0876	0.000562	0.000418	0.000461
308	971.6043	1,008.4506	39.9984	0.000559	0.000432	0.000464
309	971.6762	1,016.0327	40.0491	0.000646	0.000525	0.000529
310	971.7189	1,019.3767	40.0166	0.000553	0.000496	0.000483

**Table 4.** Error ellipses of the points of detail detected on the *Palazzo del Capitano* façade. Data refer to October 2011 in new 3D coordinate system.

**Bench Mark**	**Error Ellipses (m)**			

	**Semi-Major Axis**	**Semi-Minor Axis**	**Azimuth of Major Axis**	**Elevation**
201	0.001456	0.001422	68.82	0.001034
202	0.001445	0.001373	56.64	0.000996
203	0.001436	0.001311	54.84	0.000955
204	0.001429	0.001233	57.47	0.000908
205	0.001427	0.001173	61.52	0.000874
206	0.001422	0.001087	71.08	0.000842
207	0.001429	0.001055	76.15	0.000812
208	0.001431	0.001016	86.10	0.000794
209	0.001433	0.001010	107.19	0.000790
210	0.002422	0.001210	116.22	0.001104
211	0.001432	0.001059	119.83	0.000814
212	0.001434	0.001103	126.21	0.000836
213	0.001437	0.001153	131.51	0.000864
214	0.001443	0.001210	135.70	0.000898
301	0.001450	0.001421	72.60	0.001074
302	0.001435	0.001375	58.06	0.001045
303	0.001414	0.001269	56.18	0.000989
304	0.001406	0.001222	58.44	0.000967
305	0.001389	0.001082	72.46	0.000919
306	0.001378	0.001007	92.97	0.000903
307	0.001380	0.001017	108.28	0.000905
308	0.001384	0.001038	114.51	0.000909
309	0.001662	0.001178	128.76	0.001037
310	0.001405	0.001154	130.91	0.000946

**Table 5.** Partial and total displacements in the out of plane direction of the points of detail detected on the *Palazzo del Capitano* façade.

**Bench Mark**	**Partial Displacement 11/11–10/11 (mm)**	**Partial Displacement 12/11–11/11 (mm)**	**Partial Displacement 02/12–12/11 (mm)**	**Partial Displacement 05/12–02/12 (mm)**	**Partial Displacement 06/12–05/12 (mm)**	**Partial Displacement 10/12–06/12 (mm)**	**Total Displacement 10/12–10/11 (mm)**
201	3.60	−2.81	1.19	−1.28	7.03	−5.39	2.34
202	2.49	−2.19	0.75	−1.48	6.85	−5.33	1.09
203	2.22	−1.99	0.48	−0.22	5.48	−4.07	1.90
204	1.58	−1.51	0.90	−1.17	5.81	−4.67	0.94
205	1.77	−2.74	0.81	0.31	4.09	−2.83	1.41
206	1.23	−0.93	−0.07	0.48	---	---	1.00
207	1.38	−1.80	−0.05	1.05	3.48	−3.14	0.92
208	1.44	−1.46	0.02	0.91	1.84	-1.93	0.82
209	−0.36	−0.88	−0.77	2.33	1.26	−1.85	−0.27
210	---	---	−0.34	2.32	1.07	−1.98	0.48
211	−0.45	−0.54	−0.63	2.31	0.56	−1.86	−0.61
212	−1.13	0.47	−0.70	2.49	0.05	−0.82	0.36
213	−0.17	0.12	−0.69	3.42	−1.05	−1.18	0.45
214	−0.92	−0.30	−1.24	4.24	−1.79	0.62	0.61
301	1.60	−2.32	1.25	−1.37	7.24	−5.22	1.18
302	1.53	−1.00	0.46	−1.44	7.39	−5.41	1.53
303	2.61	−2.24	0.92	−0.22	6.16	−4.14	3.09
304	1.82	−0.12	−0.47	−0.17	5.62	−4.64	2.04
305	0.15	−0.74	0.31	−0.22	3.69	−3.27	−0.08
306	0.27	−0.95	−0.03	1.64	0.40	−2.40	−1.07
307	−0.19	−0.48	−0.06	1.80	−0.69	−1.74	−1.36
308	0.26	−1.10	−0.53	2.90	−1.21	−1.91	−1.59
309	−0.38	−2.37	1.28	3.08	−1.66	−1.23	−1.28
310	−0.58	−0.79	−1.05	3.78	−1.27	−0.67	−0.58

## References

[1] (2008). Nuove Norme Tecniche per le Costruzioni, New Technical Standards for Construction.

[2] (2008). Assessment and Reduction of the Seismic Risk of Cultural Heritage.

[3] Castagnetti C., Bertacchini E., Capra A., Dubbini M. Terrestrial Laser Scanning for Preserving Cultural Heritage: Analysis of Geometric Anomalies for Ancient Structures.

[4] Pesci A., Bonali E., Galli C., Boschi E. (2012). Laser scanning and digital imaging for the investigation of an ancient building: Palazzo d’Accursio study case (Bologna, Italy). J. Cult. Herit.

[5] Pesci A., Casula G., Boschi E. (2011). Laser scanning the Garisenda and Asinelli Towers in Bologna (Italy): Detailed deformation patterns of two ancient leaning buildings. J. Cult. Herit.

[6] Achilli V., Bragagnolo D., Fabris M., Menin A., Salemi G. Metodologie Geomatiche per il Rilievo Integrato Finalizzato alla Modellazione Strutturale.

[7] Cardaci A., Mirabella R.G., Versaci A. From the Continuos to the Discrete Model: A Laser Scanning Application to Conservation Projects.

[8] Camarda M., Guarnieri A., Milan N., Vettore A. Health Monitoring of Complex Structure Using TLS and Photogrammetry.

[9] Fabris M., Achilli V., Bragagnolo D., Menin A., Salemi G. Laser Scanning Methodology for the Structural Modelling.

[10] (2007). ABAQUS/Standard, Theory and Users Manuals, Release 6.10-1.

[11] Barbieri G., Biolzi L., Bocciarelli M., Fregonese L., Frigeri A. (2013). Assessing the seismic vulnerability of an historical building. Eng. Struct..

[12] Burland J.B., Viggiani C. (1994). Osservazioni sul comportamento della Torre di Pisa. Riv. Ital. Geotec..

[13] Giussani A., Monti C. (1985). Basilica di San Marco: Operazioni di misura per il controllo statico. Riv. Catasto dei Serv. Tec. Erar..

[14] Leica Geosystem. http://www.leica-geosystems.it/it/index.htm.

[15] Alba M., Fregonese L., Prandi F., Scaioni M., Valgoi P. Structural Monitoring of a Large Dam by Terrestrial Laser Scanning.

[16] Gordon S.J., Lichti D., Stewart M., Franke J. Structural Deformation Measurement Using Terrestrial Laser Scanners.

[17] González–Aguilera D., Gómez–Lahoz J., Sánchez J. (2008). A New approach for structural monitoring of large dams with a three-dimensional laser scanner. Sensors.

[18] Schäfer T., Weber T., Kyrinovič P., Zámečniková M. Deformation Measurement using Terrestrial Laser Scanning at the hydropower station of Gabcikovo.

[19] Lindenbergh R., Pfeifer N. A Statistical Deformation Analysis of Two Ephocs of Terrestrial Laser Data of a Lock.

[20] Alba M., Roncoroni F., Scaioni M. Application of TLS for Change Detection on Rock Faces.

[21] Scaioni M., Giussani A., Roncoroni F., Sgrenzaroli M., Vassena G. Monitoring of a Geological Sites by Laser Scanning Techniques.

[22] Van Genechten B., Demeyere T., Herinckx S., Goos J., Schueremans L., Roose D., Santana M. Terrestrial Laser Scanning in Architectural Heritage—Deformation Analysis and the Automatic Generation of 2D Cross-Sections.

[23] Alba M., Barazzetti L., Giussani A., Roncoroni F., Scaioni M. Sperimentazione di tecniche innovative per il monitoraggio delle strutture.

[24] Fassi F., Achille C., Fregonese L. (2011). Surveying and modelling the main spire of Milan Cathedral using multiple data sources. Photogramm. Rec..

[25] Achille C., Fassi F., Fregonese L. 4 Years History: From 2D to BIM for CH—The Main Spire on Milan Cathedral.

